# Artificial Intelligence-Assisted Volumetric Brain Analysis Correlated with CSF Biomarkers in Alzheimer’s Disease: A Pilot Study

**DOI:** 10.3390/diagnostics16071050

**Published:** 2026-03-31

**Authors:** Pukovisa Prawiroharjo, Amelia Nur Vidyanti, Yuliarni Syafrita, Reyhan Eddy Yunus, Aldithya Fakhri, Violine Martalia, Aileen Gabrielle, Sarah Alya Rahmayani, Gamael Marcel, Vidya Gani Wijaya, Alya Ayu Tazkia

**Affiliations:** 1 Department of Neurology, Faculty of Medicine, Universitas Indonesia, Jakarta 10430, Indonesia; 2 Department of Neurology, RSUPN Dr. Cipto Mangunkusumo, Jakarta 10320, Indonesia; 3 Department of Neurology, Rumah Sakit Universitas Indonesia, Depok 16424, Indonesia; 4 Department of Neurology, Faculty of Medicine, Public Health, and Nursing, Universitas Gadjah Mada, Yogyakarta 55281, Indonesia; amelia.nur.v@ugm.ac.id; 5 Department of Neurology, Faculty of Medicine, Universitas Andalas, West Sumatra 25127, Indonesia; syafrita.yuliarni@gmail.com; 6 Department of Radiology, Faculty of Medicine, Universitas Indonesia, Jakarta 10430, Indonesia; reyhanyunus@ui.ac.id (R.E.Y.); gamael.marcel@ui.ac.id (G.M.); 7 Faculty of Medicine, Universitas Indonesia, Jakarta 10430, Indonesia; aldithya95@gmail.com (A.F.); violinemartalia@gmail.com (V.M.); 8 Brainz Research and Innovation Center, Jakarta 12310, Indonesia; aileengabrielle1297@gmail.com (A.G.); sarahalyarahmayani@gmail.com (S.A.R.); vidyaganiwijaya@gmail.com (V.G.W.); alyatazkia27@gmail.com (A.A.T.); 9 Faculty of Medicine, Universitas Hang Tuah, Surabaya 60241, Indonesia; 10 Faculty of Medicine, Universitas Sebelas Maret, Surakarta 57126, Indonesia; 11 School of Medicine and Health Sciences, Atma Jaya Catholic University of Indonesia, Jakarta 14440, Indonesia

**Keywords:** Alzheimer’s disease, CSF biomarkers, brain volumetry, artificial intelligence, MRI, entorhinal cortex, posterior cingulate cortex, VICV

## Abstract

**Background/Objectives**: Alzheimer’s disease (AD) is a leading cause of dementia globally, yet standard diagnostic markers like cerebrospinal fluid (CSF) analysis and molecular imaging are invasive and resource-intensive. While artificial intelligence (AI)-based volumetric magnetic resonance imaging (MRI) offers a scalable and non-invasive alternative, data correlating these structural metrics with fluid biomarkers and cognitive status in Southeast Asian populations are scarce. This study addresses this critical gap by examining the within-cohort relationship between CSF biomarkers and regional brain volumes derived from AI-assisted MRI in Indonesian patients with clinically diagnosed AD, providing novel data for an underrepresented population. **Methods**: Twenty-one AD patients from three national referral hospitals in Indonesia underwent lumbar puncture for CSF biomarker analysis and 3 Tesla structural brain MRI. Brain volumes were analyzed using United Imaging Intelligence software, focusing on AD-relevant regions (hippocampus, entorhinal cortex, parahippocampus, precuneus, and posterior cingulate cortex [PCC]). **Results**: Spearman’s correlation revealed significant positive associations between CSF Aβ42 levels and several brain regions. Strong correlations were found with the right entorhinal volume indexed to intracranial volume (VICV) (r = 0.601, *p* = 0.004), right PCC VICV (r = 0.603, *p* = 0.004), right entorhinal volume (r = 0.533, *p* = 0.013), and right hippocampus VICV (r = 0.503, *p* = 0.020). Furthermore, MoCA-InA scores demonstrated highly significant positive correlations with CSF Aβ42 concentrations (r = 0.720, *p* < 0.001), right Hippocampus VICV (r = 0.703, *p* < 0.001), and right PCC VICV (r = 0.695, *p* < 0.001). No significant correlations were found between CSF pTau or the pTau/Aβ42 ratio and regional volumes. **Conclusions**: These results highlight the entorhinal cortex and PCC as early affected regions where CSF Aβ42 correlates with preserved volume, supporting their role as structural markers in early AD. The absence of pTau associations may reflect early-stage pathology or limitations of cross-sectional volumetry. In resource-limited settings, AI-assisted volumetric MRI demonstrates potential utility as a non-invasive tool for stratifying amyloid-associated brain atrophy and staging disease severity.

## 1. Introduction

Alzheimer’s disease (AD) is the most prevalent form of dementia worldwide, characterized neurobiologically by the pathological accumulation of extracellular amyloid-beta (Aβ) plaques and intracellular tau tangles, particularly within cortical regions [1]. The burden of dementia is particularly acute in Indonesia, where a recent comprehensive study estimated the prevalence at 27.9% among adults aged 65 and older, a figure significantly higher than the 4–11% prevalence reported in other Asian nations [2,3]. As a leading cause of global disability and dependence, AD imposes a profound economic strain on healthcare systems and families due to the substantial requirements for long-term care [3,4,5].

Currently, the clinical gold standard for confirming AD pathology relies on invasive cerebrospinal fluid (CSF) biomarker analysis or high-cost molecular neuroimaging, such as amyloid and tau positron emission tomography (PET). Established CSF biomarkers for AD include Aβ and tau measures, including Aβ42 and phosphorylated tau (pTau), which provide critical insights into disease pathophysiology [6,7]. However, the widespread implementation of these diagnostics in low- and middle-income countries (LMICs) is severely limited. The lumbar puncture required for CSF collection is invasive and frequently refused by patients due to discomfort or cultural stigma, while PET imaging remains prohibitively expensive and logistically inaccessible in many regions [3,4]. Consequently, there is an urgent need for accessible, non-invasive alternatives that can reliably characterize underlying pathology and disease stage.

Structural brain imaging using magnetic resonance imaging (MRI) has emerged as a non-invasive and widely accessible method for assessing neurodegeneration. Advanced computational methods, such as voxel-based morphometry and automated volumetric segmentation, enable the quantification of atrophy in AD-vulnerable structures, including the hippocampus, entorhinal cortex, and posterior cingulate cortex (PCC). While prior research has demonstrated correlations between these MRI-derived atrophy patterns and CSF biomarkers [6,7], manual or semi-automated segmentation methods can be time-consuming and prone to inter-observer variability.

The integration of artificial intelligence (AI) into medical imaging represents a paradigm shift, offering platforms capable of the automated, rapid, and reproducible quantification of brain volumes. These AI-driven tools hold the potential to detect subtle volumetric changes that may precede overt clinical symptoms, thereby facilitating more objective disease phenotyping. Despite these advancements, data correlating AI-derived brain volumetrics with established CSF biomarkers remain predominantly derived from Western cohorts. Research specifically investigating these relationships in Southeast Asian populations is scarce [8]. Addressing this knowledge gap is critical, as genetic, environmental, and lifestyle factors may influence the phenotypic expression of AD in this demographic.

Therefore, this study aims to examine the relationship between CSF biomarkers and regional brain volumes derived from an AI-assisted volumetric MRI platform in Indonesian patients. By validating the utility of AI volumetry as a surrogate for invasive biomarkers in this specific population, this research seeks to establish a scalable tool for patient stratification and monitoring for resource-limited settings.

## 2. Literature Review

### 2.1. Structural MRI and Biomarker Correlations

The relationship between fluid biomarkers and structural brain atrophy is well-documented in the literature. Neurodegeneration in AD is not uniform; it typically follows a specific topographical progression, starting in the transentorhinal region before spreading to the hippocampus and neocortex. Previous cross-sectional studies have consistently shown that lower CSF Aβ42 levels, indicative of varying amyloid plaque burden, correlate with reduced gray matter volume in the medial temporal lobe [6]. Similarly, elevated pTau levels have been associated with cortical thinning and ventricular expansion, often serving as a predictor of future cognitive decline [7]. However, the strength of these correlations varies across studies, likely due to differences in segmentation methodologies and the heterogeneity of disease stages in patient cohorts.

### 2.2. The Role of AI in Neuroimaging

Traditional volumetric analysis often relies on manual segmentation, which is labor-intensive and subject to rater bias, or older automated atlases that may struggle with anatomical variations [9]. The advent of AI and deep learning algorithms has revolutionized this field by enabling “AI-assisted volumetric analysis”. These platforms utilize deep neural networks, such as ResNet, to learn features from vast datasets, allowing for the precise segmentation of brain structures in a fraction of the time required by conventional methods [10,11]. Beyond speed, AI tools have demonstrated superior reproducibility and sensitivity in detecting subtle atrophy patterns that escape visual inspection [12]. Validating these AI tools against biological ground truths, such as CSF biomarkers, is essential for their clinical adoption [13].

### 2.3. Research Gap in Southeast Asian Populations

Despite the robust volume of AD research globally, the vast majority of biomarker and imaging studies have been conducted in North American and European populations. Emerging evidence suggests that ethnic and geographic factors influence AD risk profiles and biomarker cut-offs [3]. In Indonesia, where the prevalence of dementia is notably high, there is a paucity of data integrating advanced neuroimaging with fluid biomarkers. Most local diagnoses rely on clinical interviews and standard structural MRI without volumetric quantification. To date, no study has specifically evaluated the correlation between AI-derived volumetric metrics and CSF biomarkers in an Indonesian cohort. This study fills that critical gap, providing the first dataset linking AI volumetry with biological pathology in this underrepresented demographic.

## 3. Materials and Methods

### 3.1. Study Design

This study was designed as a cross-sectional observational analysis aiming to evaluate the correlation between brain volumetric biomarkers and CSF biomarkers in patients with AD. All data were collected at a single time point and included structural brain MRI and lumbar CSF sampling. Volumetric analysis was performed using an AI-based platform, United Imaging Intelligence (uAI^®^), to assess specific brain regions of interest (ROIs) commonly implicated in AD.

Participants were recruited from three major national referral hospitals across Indonesia: RSUP Dr. Sardjito in Yogyakarta, RSUPN Dr. Cipto Mangunkusumo in Jakarta, and RSUP Dr. M. Djamil in Padang. These centers served as representatives of the broader Indonesian population. The study population consisted of patients clinically diagnosed with AD who presented to the Memory Clinics at the respective hospitals.

### 3.2. Participants

Inclusion criteria were (1) patients who were diagnosed with AD based on Diagnostic and Statistical Manual of Mental Disorders, Fifth Edition (DSM-5) standardized clinical criteria [14], neuropsychological examination using Montreal Cognitive Assessment Indonesian Version (MoCA-InA) [15], and validated by a neurologist; (2) ability to understand and respond in Bahasa Indonesia; and (3) willingness to participate, confirmed through informed consent. Exclusion criteria included (1) history of stroke or mixed dementia; (2) other structural brain diseases such as infections, tumors, traumatic brain injury, epilepsy, or neuropsychiatric disorders such as schizophrenia or major depression; and (3) patients with contraindications to undergoing MRI.

### 3.3. Data Collection Protocol

This study included 21 patients clinically diagnosed with AD based on inclusion criteria. All participants underwent lumbar puncture for CSF collection and structural brain MRI. Detailed demographic characteristics of the 21 patients enrolled in this study are presented in Table 1. The median age of the patient cohort was 63 years, with an age range spanning from 31 to 82 years. The study population comprised a slight majority of males (12 patients, 57.1%), with females accounting for 9 patients (42.9%). Regarding educational attainment, the largest proportion of participants had completed 16 years of education (12 patients, 57.1%). This was followed by 12 years of education (6 patients, 28.6%), those with 18 years of education (2 patients, 9.5%), and one patient with 9 years of education (4.8%).

### 3.4. Ethical Considerations

The study was conducted in strict accordance with the Declaration of Helsinki and approved by the local Ethics Committee. Written informed consent was obtained from all subjects involved in the study prior to any data collection or procedures.

### 3.5. CSF Biomarker Analysis

CSF biomarker analysis was performed on lumbar CSF samples collected via standard lumbar puncture at the L3–L4 or L4–L5 interspaces. After collection, samples were centrifuged and stored at −80 °C until analysis. Biomarkers analyzed included lumbar CSF amyloid-beta 1–42 (CSF Aβ42), phosphorylated tau (CSF pTau), and the pTau/Aβ42 ratio. All measurements were conducted using commercially available enzyme-linked immunosorbent assay (ELISA) kits (Wuhan Fine Biotech Co., Ltd., Wuhan, China; Fine Test; Human Aβ42 Catalog No. EH2685, and Human Phospho-Tau T181 Catalog No. EH4701) in a certified clinical laboratory. All samples were processed in duplicate according to the manufacturer’s protocol, and intra-assay coefficients of variation (CV) were maintained within the manufacturer’s acceptable limits (<10%). While the established literature suggests ELISA-based clinical reference cutoffs of ≤700 pg/mL for Aβ42 and ≥60 pg/mL for P-Tau181 to define an AD-positive profile [16], specific dichotomous diagnostic cutoffs were not applied to stratify our cohort. Because this study utilized a correlational design, absolute continuous biomarker concentrations were utilized for all statistical analyses to maximize power and capture the spectrum of biological variability.

### 3.6. Brain MRI Acquisition Protocol and AI-Based Segmentation (uAI)

**Figure 1:**
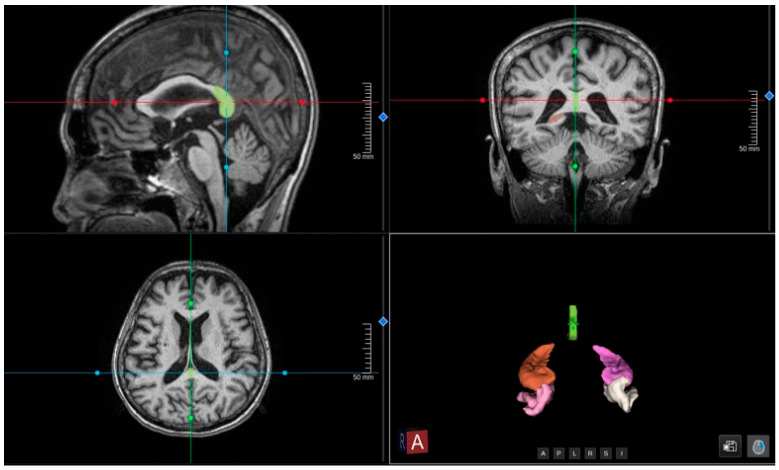
Representative visualization of AI-based segmentation using the uAI^®^ platform. Orthogonal multi-planar reconstructions (sagittal, coronal, and axial) and a 3D rendering from the 3D T1-weighted MRI show automated brain parcellation. Colored overlays indicate segmentation masks used for region-of-interest volumetric extraction. The intersecting lines indicate the multi-planar navigational axes.

Structural brain imaging was conducted using a 3 Tesla MRI scanner (Philips Healthcare, Best, The Netherlands) according to standardized neuroimaging protocols, which included a high-resolution 3D T1-weighted sequence suitable for volumetric analysis. To support accelerated scanning while maintaining image quality, image acquisition and reconstruction incorporated AI-assisted compressed sensing (ACS), which enables high-fidelity reconstruction from undersampled data. The reconstructed 3D T1-weighted images were subsequently analyzed using the United Imaging Intelligence (uAI^®^) research portal (United Imaging Intelligence, Shanghai, China) for automated volumetric assessment. This platform utilizes a deep learning architecture based on fully convolutional neural networks (e.g., ResNet-based U-Net) trained on large-scale multicenter datasets to achieve the automated, high-precision segmentation of brain structures and extraction of region-of-interest (ROI) volumes (Figure 1). This method has been shown to provide more useful and sensitive clinical accuracy in identifying neurodegeneration-related atrophy compared to standard radiological visual assessment.

### 3.7. Region of Interest (ROI) and VICV Calculation

**Figure 2:**
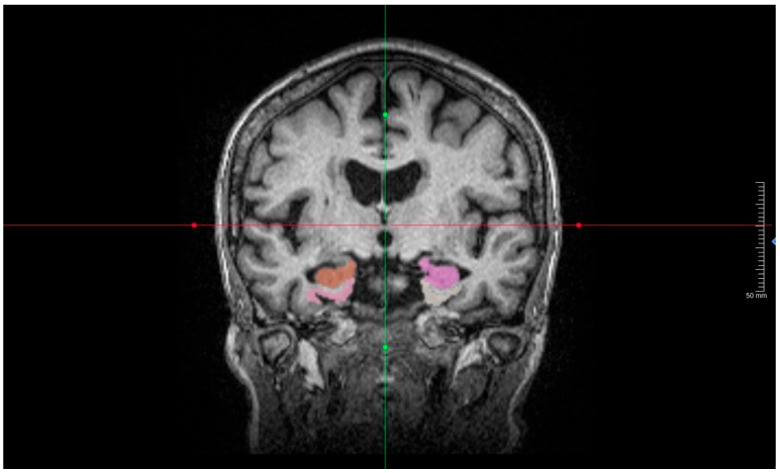
Detailed visualization of medial temporal lobe segmentation. Coronal 3D T1-weighted MRI slice demonstrating uAI^®^-derived segmentation of Alzheimer’s disease-relevant medial temporal structures. Colored overlays represent automatically segmented regions used for volumetric calculations (e.g., hippocampus and adjacent medial temporal cortex). The image is intentionally magnified to highlight the medial temporal structures, and the intersecting lines indicate the navigational axes.

ROIs were selected based on their established involvement in Alzheimer’s pathology, including the hippocampus, parahippocampal gyrus, entorhinal cortex, cuneus, precuneus, and posterior cingulate cortex (PCC) (Figure 2). For each ROI, absolute volume (V, in cm^3^), volume indexed to intracranial volume (VICV, as a percentage), and symmetry index (SI, as a percentage) were extracted. To account for inter-individual variations in head size, a critical factor in volumetric studies, we utilized the VICV. The Total Intracranial Volume (TIV) was calculated by the uAI algorithm as the sum of gray matter, white matter, and CSF volumes. The VICV for each ROI was calculated using the following formula:

(1) $$\mathrm{VICV}\;(\%)=(\frac{Absolute\;ROI\;Volume}{Total\;Intracranial\;Volume})\;\times \;100$$

In addition to absolute volumes and VICV, the SI was calculated to quantify the volumetric difference between the left and right hemispheres for each ROI. The SI is expressed as a percentage (%) and was calculated using the standard formula:

(2) $$\mathrm{SI}=(\frac{Vright-Vleft}{0.5\;\times \;(Vright+Vleft)})\;\times \;100$$

where V*right* and V*left* represent the absolute volumes of the right and left structures, respectively. SI represents the relative volume difference between the right and left hemispheres normalized to their mean bilateral volume. Positive values indicate rightward predominance, and negative values indicate leftward predominance; values near 0% indicate symmetry. All automated volumetric segmentations were visually inspected slice-by-slice. When required, manual corrections were performed by a trained neuroimaging researcher who was fully blinded to the patients’ clinical status and CSF biomarker data, adhering to established anatomical landmarks to ensure anatomical accuracy and minimize bias. Because the AI software demonstrated high robust performance, manual boundary adjustments were rare and only required in approximately <5% of the regions of interest, primarily to correct minor skull-stripping or tissue-boundary artifacts.

### 3.8. Statistical Analysis

Statistical analyses were conducted using IBM SPSS Statistics version 25. The normality of data distribution was evaluated using the Shapiro–Wilk test. Given the small sample size and non-normal distribution of most variables, non-parametric tests were applied. Specifically, Spearman’s rank correlation coefficient (ρ) was used to assess associations between the CSF biomarker levels and brain volumetric measures. All tests were two-tailed, and a *p*-value of less than 0.05 was considered statistically significant.

## 4. Results

### 4.1. CSF Biomarker Findings

The CSF biomarker levels for the 21 study participants are summarized in Table 2. The mean concentration of CSF Aβ42 was 635.20 ± 353.58 pg/mL. For CSF pTau, the mean concentration was 80.02 ± 143.93 pg/mL. The mean ratio of CSF pTau/Aβ42 was found to be 1.01 ± 4.25. While these values characterize the biomarker profile of the cohort, interrelationships between these CSF biomarkers and their correlations with brain volumes are further explored in the subsequent sections.

### 4.2. Brain Volumetric Analysis

The AI-assisted volumetric brain analysis provided quantitative measures of various regional brain volumes and their correlations with CSF biomarkers. Quantitative volumetric analysis was performed for all ROIs. Table 3 presents the descriptive statistics (Mean ± SD) for absolute volumes, VICV, and SI for the cohort.

**Figure 3:**
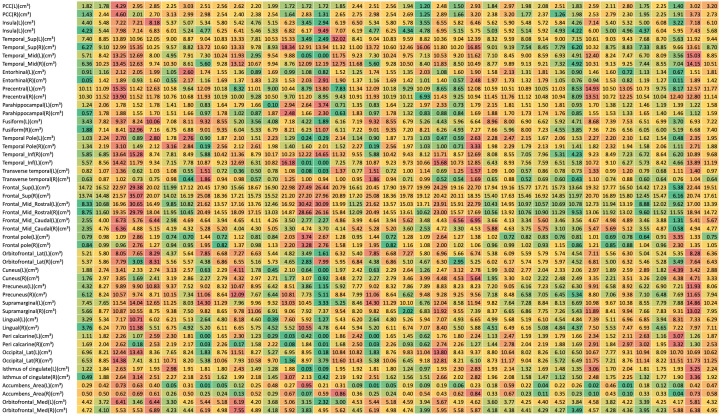
Heatmap visualization of regional brain volumes (cm^3^) across the study cohort (*n* = 21). Each column represents an individual patient, and each row represents a specific brain region. The color scale indicates the volume percentile within the cohort, where green represents lower volume and red represents higher volume. The observed heterogeneity in volumetric profiles, particularly in the hippocampus and entorhinal cortex, reflects the biological variability of disease severity within the cohort and demonstrates the sensitivity of the AI platform in characterizing individual atrophy patterns.

To visualize the variability of brain atrophy across the individual participants, a volumetric heatmap was generated (Figure 3). This highlights the heterogeneity in regions such as the hippocampus and entorhinal cortex across the cohort.

### 4.3. Correlation Analysis

A comprehensive Spearman’s correlation analysis was conducted to assess the relationships between CSF biomarkers and the total volumes of various brain regions. The analysis revealed distinct patterns of association between the CSF biomarkers and total regional brain volumes, as presented in Table 4. Significant correlations between the volumes of various brain regions are displayed in Table 5, with further details available in Supplementary Table S1.

#### 4.3.1. Aβ42 Associations

**Figure 4:**
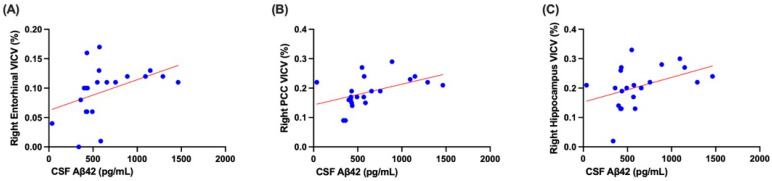
Scatter plots with linear regression lines illustrating the relationship between CSF Aβ42 levels in 21 patients and volumetric indices (VICV). (**A**) Right entorhinal cortex, (**B**) right PCC, and (**C**) right hippocampus. While regression lines are shown for visualization of trends, statistical significance was determined using Spearman’s rank correlation to account for the non-normal distribution (r = 0.601 for right entorhinal VICV, r = 0.603 for right PCC VICV, r = 0.503 for right hippocampus VICV, *p* < 0.05 for all). The blue circles represent individual patient data points, and the red lines denote the best-fit linear regression trends.

CSF Aβ42 levels demonstrated widespread, significant positive correlations with volumetric measures in AD-vulnerable regions. Strong associations were observed in the entorhinal cortex and PCC, with significant correlations also noted in the hippocampus, particularly when corrected for VICV (Figure 4). Specific findings (Table 5) indicate that CSF Aβ42 consistently showed significant positive correlations with the entorhinal cortex volumes, including left entorhinal volume (r = 0.466, *p* = 0.033), left entorhinal VICV (r = 0.486, *p* = 0.026), right entorhinal volume (r = 0.533, *p* = 0.013), and right entorhinal VICV (r = 0.601, *p* = 0.004). A significant positive correlation was also found with right hippocampus VICV (r = 0.503, *p* = 0.020). Additionally, CSF Aβ42 showed significant positive correlations with right PCC volume (r = 0.517, *p* = 0.016) and right PCC VICV (r = 0.603, *p* = 0.004).

#### 4.3.2. Phosphorylated Tau (pTau) and Symmetry Index

In contrast to amyloid markers, neither CSF pTau nor the CSF pTau/Aβ42 ratio demonstrated any statistically significant correlations (*p* < 0.05) with any regional brain volumes or VICV measures in this cohort (Table 4). Furthermore, the SI calculated to assess lateralization of atrophy showed no significant correlation with any CSF biomarkers. As detailed in Supplementary Table S1, the SI for the hippocampus, entorhinal cortex, and PCC did not exhibit statistically significant associations (*p* > 0.05). These comprehensive correlation findings underscore the differential relationships between specific CSF biomarkers and distinct regional brain volumes in the context of AD.

### 4.4. Clinical and Cognitive Correlations

To evaluate the clinical relevance of the imaging and fluid biomarkers, Spearman’s rank correlation was performed using the patients’ Montreal Cognitive Assessment Indonesian Version (MoCA-InA) scores. MoCA-InA scores demonstrated a highly significant positive correlation with the CSF Aβ42 concentrations (r = 0.720, *p* < 0.001). Furthermore, global cognitive scores showed robust positive correlations with specific volumetric indices, notably the right hippocampus VICV (r = 0.703, *p* < 0.001), right PCC VICV (r = 0.695, *p* < 0.001), and right PCC absolute volume (r = 0.625, *p* = 0.002). There was no statistically significant correlation observed between MoCA-InA scores and entorhinal volumes, likely reflecting the global nature of the MoCA assessment compared to the highly specific, early episodic memory functions localized to the entorhinal cortex. The complete correlation findings are summarized in Table 6.

## 5. Discussion

### 5.1. Summary of Key Findings

This study explored the relationship between CSF biomarkers and AI-assisted volumetric brain measurements in a clinically diagnosed cohort of AD patients from three national referral hospitals in Indonesia. The primary objective was to investigate within-cohort associations between biological markers of pathology and structural phenotypic expression. Using a deep learning-based MRI segmentation platform (uAI^®^), quantitative volumes of key AD-related brain regions were assessed and compared with levels of CSF Aβ42, pTau, and the pTau/Aβ42 ratio to evaluate their potential utility for patient stratification and clinical staging.

The primary finding was a significant and consistent positive correlation between CSF Aβ42 concentrations and volumes of multiple brain regions implicated in AD pathology. Notably, strong correlations were observed in both the right and left entorhinal cortices, with the right entorhinal VICV showing the highest correlation (r = 0.601, *p* = 0.004), followed by the right PCC VICV (r = 0.603, *p* = 0.004), right entorhinal volume (r = 0.533, *p* = 0.013), and right hippocampus VICV (r = 0.503, *p* = 0.020). These findings also underscore the relevance of intracranial volume-corrected measures (VICV), which frequently exhibited stronger correlations with the CSF Aβ42 levels compared to the raw regional volumes, unilateral volumes, or symmetry indices. This suggests that VICV provides a more accurate representation of true brain atrophy by accounting for individual head size variations [17].

However, our study did not find significant correlations between CSF pTau levels and regional brain volumes. While some studies suggest that CSF pTau predicts ongoing neurodegeneration, the relationship can be variable [18,19]. It is also notable that the most robust associations with Aβ42 were localized to right-sided entorhinal and PCC regions, possibly reflecting lateralized vulnerability [19]. Given the small sample size, this observation should be considered strictly hypothesis-generating and requires validation in larger cohorts.

Overall, these findings highlight the relevance of CSF Aβ42 as a marker of structural brain integrity and support the use of AI-assisted volumetric MRI as a feasible and non-invasive tool to detect and quantify region-specific brain atrophy in AD. The results provide preliminary evidence that such imaging biomarkers may serve as surrogate indicators of underlying amyloid pathology, particularly in resource-limited settings where lumbar puncture or molecular imaging may be less accessible.

### 5.2. Pathophysiological Interpretation

The entorhinal cortex is one of the earliest and most vulnerable regions affected in AD due to its central role in memory and spatial navigation [20,21]. AD pathology typically begins with Aβ accumulation and pTau formation, with the entorhinal cortex among the first regions to exhibit neurodegeneration. The significant positive correlation observed in our cohort between CSF Aβ42 and entorhinal volume requires both biological and methodological consideration. Biologically, decreased CSF Aβ42 reflects the pathological sequestration of amyloid into extracellular plaques within the brain [6,13]. This amyloid accumulation is not inert; it triggers a localized cascade of neurotoxicity, neuroinflammation, and synaptic dysfunction that eventually manifests as macroscopic neuronal loss [13]. The entorhinal cortex is highly susceptible to this early amyloid-driven cascade [21]. Therefore, lower CSF Aβ42 (reflecting higher brain plaque burden) directly mirrors the reduced entorhinal volume observed, as volume loss may exceed that of the hippocampus early in the disease spectrum [20,21]. Methodologically, identifying this correlation within a relatively small cohort highlights the precision of advanced neuroimaging. The anatomical boundaries of the entorhinal cortex are highly complex, making traditional manual segmentation highly susceptible to inter-rater variability and noise [9]. The robust correlation observed in this study is likely unmasked by the automated precision of the deep learning-based uAI platform [10,11]. Furthermore, the correlation was strongest when utilizing the right entorhinal volume indexed to intracranial volume (VICV). Normalizing the absolute AI-derived volume to the total intracranial volume mathematically eliminates the confounding variable of individual head size variations, allowing the true biological relationship between amyloid toxicity and structural atrophy to reach statistical significance [17].

The PCC, a key region of the default mode network, is also a critical site of structural and metabolic decline in AD. Reduced CSF Aβ42 levels have been shown to correlate with PCC atrophy, reflecting amyloid-related network disruption. Given its strong connectivity with the hippocampus and entorhinal cortex, the PCC plays a critical role in memory circuits. Our finding of a significant correlation between CSF Aβ42 and PCC volume is consistent with this pattern and supports its utility as a marker of AD-related neurodegeneration [22].

The addition of MoCA-InA scores further contextualizes these structural findings. The strong correlations between global cognitive scores, CSF Aβ42, and larger structures like the hippocampus and PCC align with the expected trajectory of cognitive decline as neurodegeneration spreads [17]. The lack of a significant linear correlation between the MoCA-InA score and the entorhinal cortex may underscore the focal nature of early entorhinal damage [20,21]; while structural loss here correlates strongly with biological amyloid burden, it may not yet fully manifest on a global cognitive screening instrument that heavily weighs broad executive and visuospatial domains [15].

The absence of significant correlations between CSF pTau or the pTau/Aβ42 ratio and regional brain volumes in this cohort warrants careful interpretation. It is possible that tau pathology did not yet result in detectable macrostructural atrophy in our specific sample [17]. Tau-associated neurodegeneration often manifests initially as subtle cortical thinning or microstructural white matter changes rather than overt volumetric loss measurable by conventional MRI volumetry [23]. Thus, tau pathology may contribute to neurodegeneration in a spatially heterogeneous manner better captured by cortical thickness measures or diffusion tensor imaging (DTI) or tau PET imaging rather than gross volumetric assessments [24].

However, methodological limitations must also be considered. The relatively small sample size and cross-sectional design reduce statistical power to detect tau-related neurodegenerative effects, which are known to evolve dynamically over time [18]. In addition, volumetric MRI analyses typically assess total regional volume, which may overlook focal tau-associated damage confined to specific cortical layers or white matter tracts. This spatial heterogeneity and focal distribution of tau pathology require high-resolution or multimodal imaging techniques for accurate characterization. Furthermore, although our analysis incorporated global cognitive staging (MoCA-InA), the lack of significant correlation with pTau levels suggests that the absence of structural associations may be attributable to the specific clinical stage of our cohort, prior to the onset of widespread, tau-induced macro-atrophy or to the sensitivity limitations of gross volumetric averaging [24].

### 5.3. Role of AI-Assisted Volumetric MRI

In our workflow, ACS refers to the AI-enabled acquisition/reconstruction approach that accelerates MRI while maintaining image quality, whereas uAI volumetry refers to the post-processing step that performs automated segmentation and volume extraction from the reconstructed images. ACS uses deep neural networks like ResNet to learn features from high-quality, fully sampled images and reconstruct high-fidelity images from undersampled raw data [10,25]. ACS integrates compressed sensing, parallel imaging, and deep learning to reduce artifacts and enhance image quality. In contrast, automated brain structure segmentation for Alzheimer’s evaluation is performed during the uAI volumetry step, using the ACS-reconstructed images as input. It enables automated brain structure segmentation for Alzheimer’s evaluation and allows accelerated high-resolution 3D MRI, achieving 29–50% faster acquisition without sacrificing image quality [11,25].

In this study, we used AI-assisted volumetric analysis software (Computer-Aided Analysis System for Brain Parcellation, version R001) from United Imaging Intelligence integrated with their 3 Tesla MRI system. The platform employs ACS to accelerate acquisition and reconstruct high-resolution images with preserved anatomical detail. Prior validation showed ACS reduced scan times by 33–44% for T1- and T2-weighted sequences while maintaining or improving SNR and CNR [11]. This enabled rapid, reproducible quantification of Alzheimer’s-related structures for correlation with CSF biomarkers.

Beyond detection, AI volumetry supports prognostic evaluation. A comparative study between automated systems (NeuroQuant, Siemens Morphometry, icobrain dm) and traditional visual MTA ratings showed that volumetric ratios (e.g., inferior lateral ventricle/hippocampus) closely matched expert scoring and improved sensitivity to subtle atrophy [12]. The volumetric heterogeneity observed in our cohort (Figure 3) further highlights the individual variability in AD progression. This underscores the necessity for precision diagnostics, such as AI-assisted volumetry, which can quantify these subtle inter-individual differences more reliably than visual inspection alone.

### 5.4. Clinical Implications

ACS represents a significant advancement in MRI, enabling the faster acquisition of high-resolution brain images with reduced noise and artifacts. This capability is particularly valuable in the context of AD, where the accurate detection of structural changes, such as hippocampal and medial temporal lobe atrophy, is critical for patient stratification and disease monitoring [11,25].

ACS enhances the visualization of cortical thinning and white matter loss, supporting detailed disease characterization. Rapid acquisition improves patient compliance and reduces motion artifacts—crucial in elderly or cognitively impaired populations. Moreover, by preserving volumetric accuracy, ACS allows for the reliable assessment of brain atrophy in relation to cerebrospinal fluid biomarkers such as Aβ42 and phosphorylated tau, supporting multimodal phenotyping of AD [11,26].

In research settings, shorter scan times improve data consistency and enable more frequent follow-up imaging, facilitating longitudinal monitoring of disease progression and treatment response. While direct studies in AD are limited, findings from neurological imaging confirm that ACS improves image quality and acquisition efficiency, supporting its broader use in both clinical and research applications [11].

### 5.5. Limitations

It is important to acknowledge that this study has several limitations. First, the sample size was small (*n* = 21), and the study design did not include a cognitively normal control group. We acknowledge that this limits diagnostic discrimination (distinguishing AD from normal aging); however, the primary aim of this study was to investigate within-cohort associations between biomarkers and structural phenotypes. For such correlational analyses, a control group is not strictly required to observe how biological variability covaries with neuroanatomical measures. Furthermore, obtaining CSF samples from healthy individuals poses significant ethical and logistical challenges in our local healthcare context. Additionally, the cross-sectional design inherently prevents causal inference. The lack of significant correlations with pTau may reflect limited statistical power, disease stage heterogeneity within the cohort, or methodological constraints of gross volumetry rather than a true biological dissociation. Second, while we established strong correlations between imaging markers, fluid biomarkers, and general cognitive status (MoCA-InA), linear correlations with granular neuropsychological domains (e.g., specific memory recall versus executive function) were not performed. Future studies should integrate detailed neuropsychological domain testing to explore the precise functional impact of the observed region-specific volumetric changes. Third, given the exploratory nature of this pilot study and the small sample size, formal corrections for multiple comparisons (e.g., false discovery rate) were not applied to the ROI-level correlations. While we acknowledge that this increases the risk of Type I errors, applying strict corrections at this pilot scale would severely limit the detection of biologically relevant signals. Consequently, the regional significance reported here should be interpreted conservatively as exploratory and hypothesis-generating, warranting validation in larger cohorts before broader generalization.

### 5.6. Further Directions

Future research should aim to address the limitations of this study. It is imperative to conduct studies with larger sample sizes to enhance statistical power and the generalizability of findings. While challenging, navigating the ethical framework to include a control group of cognitively normal individuals would validate disease specificity. Future studies should incorporate detailed cognitive staging and functional measures alongside global screening tools like MoCA to improve the clinical interpretability of structural changes. Longitudinal studies are also crucial to track disease progression and the dynamic changes in brain volumes and biomarker levels over time.

## 6. Conclusions

In this study, we found significant correlations between CSF Aβ42 levels and regional brain volumes in areas commonly affected by AD, such as the entorhinal cortex, hippocampus, and posterior cingulate cortex. Notably, the use of intracranial volume-corrected measures (VICV) often demonstrated stronger correlations with CSF biomarkers compared to absolute volumes, unilateral volumes, or asymmetry indices, highlighting the utility of VICV for a more robust assessment of amyloid-related brain atrophy. Furthermore, the integration of global cognitive scores (MoCA-InA) revealed strong clinical correlations with fluid amyloid burden and volumetric loss in the hippocampus and posterior cingulate cortex. These results suggest that AI-assisted volumetric MRI, particularly when utilizing VICV and combined with cognitive screening, shows preliminary potential as a non-invasive marker for reflecting amyloid-associated pathology and clinical severity in Alzheimer’s disease; however, conservative interpretation is warranted at this pilot scale. Ultimately, the statistical limitations, small sample size, and absence of a cognitively normal control group limit the broader generalizability of these findings. Nevertheless, the strong within-cohort associations observed in this underrepresented population support the continued investigation of AI-volumetry as a tool for clinical stratification, especially in resource-limited settings where CSF testing is less accessible.

## Figures and Tables

**Table 1:** Demographic characteristics of the study cohort (*n* = 21 patients).

Characteristics	Value (*n* = 21)
Age (median [min–max])	63 (31–82)
Gender (*n* [%])	
Female	9 (42.9)
Male	12 (57.1)
Years of Education (*n* [%])	
9	1 (4.8)
12	6 (28.6)
16	12 (57.1)
18	2 (9.5)

**Table 2:** Summary of cerebrospinal fluid (CSF) biomarker concentrations.

Characteristics	Value (*n* = 21) (Mean ± SD)
CSF Aβ42	635.20 ± 353.58
CSF pTau	80.02 ± 143.93
CSF pTau/Aβ42 Ratio	1.01 ± 4.25

**Table 3:** Descriptive statistics of regional brain volumes, VICV, and SI (*n* = 21).

Region of Interest (ROI)	Absolute Volume (cm^3^) (Mean ± SD)	VICV (%) (Mean ± SD)	Symmetry Index (%) (Mean ± SD)
Hippocampus			
Left	2.74 ± 0.97	0.20 ± 0.07	−0.06 ± 0.53
Right	2.83 ± 1.05	0.21 ± 0.07	
Parahippocampus			
Left	1.54 ± 0.31	0.12 ± 0.03	−0.03 ± 0.19
Right	1.51 ± 0.33	0.11 ± 0.03	
Entorhinal			
Left	1.47 ± 0.50	0.11 ± 0.04	0.25 ± 0.51
Right	1.27 ± 0.58	0.09 ± 0.04	
Cuneus			
Left	2.56 ± 0.81	0.19 ± 0.06	−0.17 ± 0.36
Right	3.00 ± 0.79	0.22 ± 0.06	
Precuneus			
Left	7.59 ± 2.26	0.55 ± 0.13	−0.08 ± 0.32
Right	8.00 ± 1.61	0.59 ± 0.12	
PCC			
Left	2.32 ± 0.79	0.17 ± 0.05	−0.10 ± 0.38
Right	2.54 ± 0.76	0.19 ± 0.05	

Note: SD = Standard Deviation.

**Table 4:** Correlation coefficients between CSF biomarkers and total regional brain volumes.

Component	CSF Aβ42		CSF pTau		CSF pTau/Aβ42 Ratio	
	Correlation Coefficient	*p*-Value	Correlation Coefficient	*p*-Value	Correlation Coefficient	*p*-Value
Hippocampus Volume	0.300	0.186	0.029	0.902	−0.258	0.258
Hippocampus VICV	0.409	0.066	0.022	0.924	−0.313	0.167
Hippocampus Symmetry Index	−0.163	0.480	−0.144	0.533	−0.066	0.775
Parahippocampus Volume	0.102	0.660	0.162	0.482	0.165	0.475
Parahippocampus VICV	0.225	0.327	−0.003	0.991	−0.060	0.797
Parahippocampus Symmetry Index	−0.140	0.546	−0.132	0.567	0.022	0.924
Entorhinal Volume	0.516 *	0.017	−0.038	0.871	−0.334	0.139
Entorhinal VICV	0.513 *	0.018	−0.026	0.911	−0.336	0.136
Entorhinal Symmetry Index	−0.284	0.212	−0.181	0.434	−0.126	0.586
Cuneus Volume	0.277	0.224	−0.022	0.924	−0.234	0.308
Cuneus VICV	0.385	0.085	−0.044	0.849	−0.305	0.179
Cuneus Symmetry Index	−0.418	0.059	−0.013	0.955	0.306	0.177
Precuneus Volume	0.335	0.138	0.240	0.294	−0.045	0.845
Precuneus VICV	0.385	0.085	0.273	0.232	−0.073	0.754
Precuneus Symmetry Index	0.251	0.273	0.366	0.103	0.116	0.618
PCC Volume	0.487 *	0.025	0.261	0.253	−0.203	0.378
PCC VICV	0.624 **	0.002	0.268	0.241	−0.290	0.203
PCC Symmetry Index	−0.193	0.402	−0.126	0.586	0.012	0.960

Note: ** Correlation is significant at the 0.01 level (2-tailed). * Correlation is significant at the 0.05 level (2-tailed).

**Table 5:** Correlation coefficients between CSF biomarkers and specific regional brain volumes.

Component	CSF Aβ42		CSF pTau		CSF pTau/Aβ42 Ratio	
	Correlation Coefficient	*p*-Value	Correlation Coefficient	*p*-Value	Correlation Coefficient	*p*-Value
Right Hippocampus VICV	0.503 *	0.020	0.058	0.801	−0.329	0.146
Left Entorhinal Volume	0.466 *	0.033	−0.058	0.801	−0.377	0.092
Left Entorhinal VICV	0.486 *	0.026	−0.074	0.750	−0.410	0.065
Right Entorhinal Volume	0.533 *	0.013	−0.010	0.964	−0.266	0.243
Right Entorhinal VICV	0.601 **	0.004	−0.036	0.876	−0.344	0.127
Right PCC Volume	0.517 *	0.016	0.153	0.507	−0.297	0.190
Right PCC VICV	0.603 **	0.004	0.083	0.720	−0.364	0.105

Note: ** Correlation is significant at the 0.01 level (2-tailed). * Correlation is significant at the 0.05 level (2-tailed).

**Table 6:** Correlation coefficients between MoCA-InA scores, CSF biomarkers, and specific regional brain volumes.

Variables	Correlation Coefficient (r)	*p*-Value
CSF Biomarkers		
CSF Aβ42	0.720 **	<0.001
CSF pTau	0.089	0.701
CSF pTau/Aβ42 Ratio	−0.397	0.074
Regional Brain Volumes		
Right Hippocampus VICV	0.703 **	<0.001
Left Entorhinal Volume	0.336	0.137
Left Entorhinal VICV	0.361	0.108
Right Entorhinal Volume	0.317	0.162
Right Entorhinal VICV	0.407	0.067
Right PCC Volume	0.625 **	0.002
Right PCC VICV	0.695 **	<0.001

Note: ** Correlation is significant at the 0.01 level (2-tailed).

## Data Availability

The data presented in this study are available on request from the corresponding author. The data are not publicly available due to privacy restrictions regarding patient medical records.

## Supplementary Materials

The following supporting information can be downloaded at: https://www.mdpi.com/article/10.3390/diagnostics16071050/s1, Table S1: Correlation coefficients between CSF biomarkers and regional brain volumes.

## Abbreviations

The following abbreviations are used in this manuscript:

Aβ	Amyloid-beta
ACS	AI-assisted compressed sensing
AD	Alzheimer’s disease
AI	Artificial intelligence
CSF	Cerebrospinal fluid
CNR	Contrast-to-noise ratio
DSM-5	Diagnostic and Statistical Manual of Mental Disorders, Fifth Edition
DTI	Diffusion tensor imaging
ELISA	Enzyme-linked immunosorbent assay
LMICs	Low- and middle-income countries
MoCA-InA	Montreal Cognitive Assessment—Indonesian Version
MRI	Magnetic resonance imaging
MTA	Medial temporal atrophy
PCC	Posterior cingulate cortex
PET	Positron emission tomography
pTau	Phosphorylated tau
ROI	Region of interest
SNR	Signal-to-noise ratio
TIV	Total intracranial volume
uAI	United Imaging Intelligence
VICV	Volume indexed to intracranial volume
