# Supplementary material for: Artificial Intelligence-Assisted Volumetric Brain Analysis Correlated with CSF Biomarkers in Alzheimer’s Disease: A Pilot Study
Source: Diagnostics (Basel). 2026 Mar 31;16(7):1050. doi: 10.3390/diagnostics16071050 (PMC13073183; doi:10.3390/diagnostics16071050)
Supplement: Supplementary file 1 [file diagnostics-16-01050-s001.zip › diagnostics-4193158-supplementary.pdf]

Supplementary Table S1. Correlation Coefficients Between CSF Biomarkers and Regional Brain Volumes

Component	**CSF Aβ42**		**CSF pTau**		**Ratio CSF pTau/Aβ42**	
	Correlation coefficient	P-value	Correlation coefficient	P-value	Correlation coefficient	P-value
Left Hippocampus Volume	.129	.577	-.129	.579	-.275	.227
Left Hippocampus VICV	.088	.705	-.145	.529	-.270	.236
Symmetry Index	-.163	.480	-.144	.533	-.066	.775
Right Hippocampus Volume	.379	.090	.065	.780	-.268	.241
Right Hippocampus VICV	.503*	.020	.058	.801	-.329	.146
Left Parahippocampus Volume	.059	.799	.074	.750	.101	.662
Left Parahippocampus VICV	.196	.394	-.009	.969	-.051	.827
Symmetry Index	-.140	.546	-.132	.567	.022	.924
Right Parahippocampus Volume	.130	.575	.143	.537	.160	.489
Right Parahippocampus VICV	.176	.445	.062	.788	.051	.827
Left Entorhinal Volume	.466*	.033	-.058	.801	-.377	.092
Left Entorhinal VICV	.486*	.026	-.074	.750	-.410	.065
Symmetry Index	-.284	.212	-.181	.434	-.126	.586
Right Entorhinal Volume	.533*	.013	-.010	.964	-.266	.243
Right Entorhinal VICV	.601**	.004	-.036	.876	-.344	.127
Left Cuneus Volume	.167	.470	.027	.907	-.123	.594
Left Cuneus VICV	.196	.394	.145	.529	-.062	.788
Symmetry Index	-.418	.059	-.013	.955	.306	.177
Right Cuneus Volume	.322	.154	-.243	.289	-.391	.080
Right Cuneus VICV	.377	.092	-.251	.273	-.429	.053
Left Precuneus Volume	.310	.171	.292	.199	.003	.991
Left Precuneus VICV	.373	.095	.408	.067	.014	.951
Symmetry Index	.251	.273	.366	.103	.116	.618
Right Precuneus Volume	.284	.212	.019	.933	-.179	.437
Right Precuneus VICV	.406	.068	.023	.920	-.262	.251
Left PCC Volume	.388	.082	.097	.674	-.269	.239
Left PCC VICV	.428	.053	.158	.493	-.264	.248
Symmetry Index	-.193	.402	-.126	.586	.012	.960
Right PCC Volume	.517*	.016	.153	.507	-.297	.190
Right PCC VICV	.603**	.004	.083	.720	-.364	.105

Note: **. *Correlation is significant at the 0.01 level (2-tailed).*
**. Correlation is significant at the 0.05 level (2-tailed)*
